# Glial interference: impact of type I interferon in neurodegenerative diseases

**DOI:** 10.1186/s13024-022-00583-3

**Published:** 2022-11-26

**Authors:** Ethan Roy, Wei Cao

**Affiliations:** Department of Anesthesiology, Critical Care and Pain Medicine, McGovern Medical School, University of Texas Health Science Center at Houston, Houston, TX 77030 USA

Reactive microglia accompany many neurodegenerative conditions and their contribution to disease progression is increasingly appreciated. Despite being a minor cell population in the brain, microglia are by no means homogeneous. In recent years, single-cell RNA sequencing analyses have identified multiple subpopulations of these cells in brains harboring Alzheimer’s disease (AD) pathology. Among them is a subset coined interferon response microglia (IRM) [1], which not only emerge in response to Aβ plaques but also spontaneously in aged brain [2].

Type I interferon (IFN-I) is an innate cytokine family produced in response to viral infections as a first line of defense for the host. Engagement of IFN-I with its cognate receptor leads to the transcription of a large panel of IFN-I-stimulated genes (ISGs) that operate in concert to restrict viral replication. Experimentally these ISGs serve as a molecular signature for the activity of IFN-I in cells and tissues. IFN-I production is tightly regulated to prevent pathology, as patients with persistent elevated IFN-I manifest autoinflammatory diseases that affect multiple organs including the brain [3].

Recognition of microbial or self nucleic acids by innate immune sensors drives an immune response leading to the production of IFN-I and proinflammatory mediators. In the brains of human AD and mouse models alike, a significant portion of Aβ plaques contain DNA and/or RNA [4]. Microglia are highly activated by these complex plaques and some adopt IRM phenotype, upregulating numerous ISGs [4, 5]. A fate-mapping experiment revealed an age-dependent accumulation of IRM immediately adjacent to Aβ plaques, underscoring a chronic IFN activation in AD brain [6].

IFN-I has profound effects on all mammalian cells, which is particularly true in brain. It can induce the expression of interferon induced transmembrane protein 3 (IFITM3) in neurons and astrocytes, which boosts γ-secretase activity to promote amyloid precursor protein (APP) cleavage thus increasing amyloid pathology [7]. Additionally, β-secretase expression is regulated by interferon and STAT1, the latter mediating signaling proximal to IFN-I receptor. Consequently, ablation of IFN-I receptor gene selectively from neural cells reduces Aβ plaques in a mouse AD model [6], affirming a feedforward loop whereby plaque-induced inflammation further exacerbates AD pathology.

Tonic IFN-I signaling critically modulates peripheral biology in many aspects. Likewise in the brain, basal steady-state IFN-I signaling prevents spontaneous protein aggregation in neurons [8]. Developing brain also utilizes STAT1 to facilitate selective removal of inactive pre-synaptic terminals during activity-dependent synapse refinement [9]. In AD brain, elevated IFN-I response to Aβ plaques stimulates neural STAT1 activity to induce pathogenic pre-synaptic loss [6].

Plaque-associated microglia represent a central player driving neuroinflammation in AD. While phagocytosing Aβ, entrapping parenchymal plaques and sealing off Aβ oligomers, these cells nevertheless participate in disease progression by releasing proinflammatory factors and eliminating synapses pathologically. On one hand, extracellular IFN-I produced by them sustains a highly activated state of microglia, as IFN-I receptor blockade effectively reduced the extend of microgliosis [4, 6]; on the other hand, IRMs excessively engulf post-synaptic structures that results in the loss of functional synapses. Long-term blockade of IFN-I receptor in vivo effectively rescues the memory and behavioral deficits of AD mice, which is accompanied by reduced synaptic deficits, microgliosis, inflammation, and neuritic pathology [6].

The most common genetic developmental disorder, Down syndrome (DS) patients frequently develop early onset AD. DS is caused by trisomy of human chromosome 21, which harbors not only *APP* but also *IFNAR1* and *IFNAR2* that encode both IFN-I receptor subunits. Consequently, DS patients display increased systemic interferon response [10]. Using hiPSC-based organoid and chimeric mouse models, Jin et al. recently showed that DS microglia prominently display IRM phenotype and over-prune synapses, both of which can be alleviated by knocking down *IFNAR* genes [11].

A major risk factor for frontotemporal degeneration and amyotrophic lateral sclerosis is hexanucleotide repeat expansion in *C9orf72*, which is also associated with AD. Intriguingly, C9orf72 deficiency leads to the overexpression of IFN-I in myeloid cells and microglia through cGAS-STING, a cytosolic DNA sensing pathway. Microglia lacking C9orf72 assume IRM phenotype and perform enhanced synaptic pruning, exacerbating neuronal dysfunction and learning and memory deficits in a mouse model of AD [12].

In Parkinson’s disease (PD), α-synuclein aggregates promote the pathologic development of the disease. αSyn fibrils induce DNA damage response in microglia and activate the cGAS-STING pathway, leading to an innate IFN-I response. Importantly, microglial STING activation drives both neuroinflammation and neurodegeneration in a mouse model of α-synucleinopathy [13]. Independently, Parkin and PINK1, two genetic risk factors for PD, restrain STING activation by facilitating mitophagy, whereas mice deficient in these genes are susceptible to excessive IFN-I signaling in the periphery [14]. However, how microglia lacking Parkin or PINK1 operate in the brain is unknown.

These studies collectively highlight an emerging theme of pathogenic participation by microglia undergoing aberrant IFN-I response in neurodegeneration (Fig. 1). However, deeper cellular and molecular dissection is necessary for effective clinical translation. First, we need a better understanding of whether IFN-I directly mediates neuronal loss and brain atrophy, along with synaptic damage, in diseased brain. Particularly important in AD is to elucidate the impact of IFN-I signaling on the neuropathologies associated with neurofibrillary tangles, which affect disease progression more profoundly than amyloid plaques. Second, we should identify key pathologic executors among ISGs to fully comprehend how IFN-I response drives neurodegeneration. A good example is IFITM3 in facilitating Aβ biogenesis, as described earlier [7]; another is Axl, a member of the TAM receptor kinase family, which is implicated in IRM-mediated synapse elimination [6]. Moreover, the functional and evolutionary trajectory of IRM requires further delineation. Microglia subsets largely correspond to various activation states. Whether IRM can revert to homeostasis or switch to other inflammatory statuses remains to be assessed. Progression of IRM to a senescent state by DS-derived microglia [11] implies a potential dark fate of these cells. Further, we do not yet have a grasp on the molecular basis for the generation of IRM during brain aging. It is particularly important to examine how crucial these cells are to various neurodegenerative processes that plague elderly and genetically predisposed populations.

**Fig. 1:**
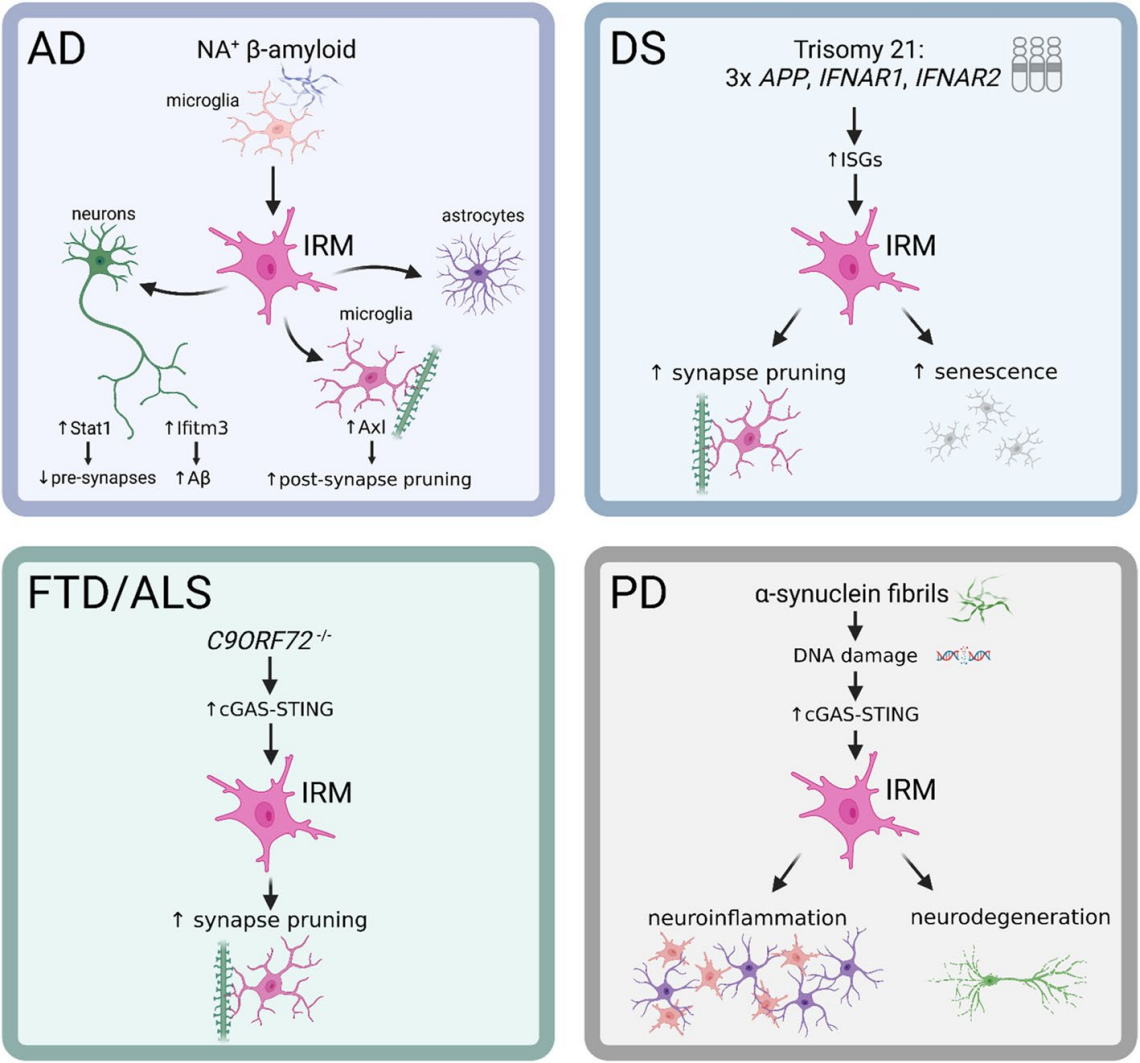
Schematic illustrating the central role of interferon-response microglia (IRM) in pathophysiology of several prominent neurodegenerative disorders. This unique cell type arises through distinct etiologies in each disease, but pathological sequelae resulting from their activation follow the common themes of damage to neurons (synapse alterations and neuronal loss) and neuroinflammation. NA^+^ amyloid = nucleic acid-containing amyloid. Created with BioRender.com

Besides IRMs, a subset of astrocytes harboring the signature of the interferon response has been identified from large-scale single-cell profiling and in vivo IFN-I reporter labeling under neurodegenerative conditions [5, 13]. The functional impact of these cells on brain pathology requires further investigation. Of note, IFNγ, i.e. type II interferon (IFN-II), triggers JAK/STAT signaling and the expression of genes that have significant overlap with ISGs induced by IFN-I. In the Aβ model, blocking IFN-II did not elicit the protection observed with IFN-I blockade, whereas neural *Ifnar1* deletion was sufficient to diminish aberrant IFITM3 activity [3, 5], suggesting preferential involvement of IFN-I under AD-related condition. Nevertheless, the IFN-II receptor gene dose is similarly elevated as that of IFNARs in DS [10], and IFN-II was shown to counteract the pathogenic effect of IFN-I in aging brain [15]. Hence, it is important to dissect the relative contributions of IFN families to various neuropathologies. In summary, this is an exciting era of neuroinflammation research and tremendous development on this topic is sure to come.

## Data Availability

Not applicable. The study contains publicly available data from published studies.
